# Fascicle-selective kilohertz-frequency neural conduction block with longitudinal intrafascicular electrodes

**DOI:** 10.1088/1741-2552/adc62a

**Published:** 2025-04-04

**Authors:** Louis Regnacq, Anil K Thota, Arianna Ortega Sanabria, Laura McPherson, Sylvie Renaud, Olivier Romain, Yannick Bornat, James J Abbas, Ranu Jung, Florian Kölbl

**Affiliations:** 1 ETIS Lab, UMR 8051, CY Cergy Paris University, ENSEA, Cergy, France; 2 IMS Lab, UMR 5218, University Bordeaux, Bordeaux INP, Talence, France; 3 The Institute for Integrative & Innovative Research—I3R,University of Arkansas, Fayetteville, AR, United States of America; 4 Department of Neurology, Washington University, St. Louis, MO, United States of America

**Keywords:** peripheral nerve stimulation, conduction block, kilohertz frequency stimulation, longitudinal intrafascicular electrodes

## Abstract

*Objective.* Electrical stimulation of peripheral nerves is used to treat a variety of disorders and conditions. While conventional biphasic pulse stimulation typically induces neural activity in fibers, kilohertz (kHz) continuous stimulation can block neural conduction, offering a promising alternative to drug-based therapies for alleviating abnormal neural activity. This study explores strategies to enhance the selectivity and control of high-frequency neural conduction block using intrafascicular electrodes. *Approach. In vivo* experiments were conducted in a rodent model to assess the effects of kHz stimulation delivered via longitudinal intrafascicular electrodes (LIFEs) on motor axons within the tibial and common peroneal fascicles of the sciatic nerve. *Main results.* We demonstrated that a progressive and selective block of neural conduction is achievable with LIFEs. We showed that the amount of neural conduction block can be tuned by adjusting the amplitude and frequency of kHz stimulation. Additionally, we achieved interfascicular selectivity with intrafascicular electrodes, with this selectivity being modulated by the kHz stimulation frequency. We also observed a small amount of onset response spillover, which could be minimized by increasing the blocking stimulus frequency. Muscle fatigue was quantified during kHz continuous stimulation and compared to control scenarios, revealing that the muscle was able to recover from fatigue during the block, confirming a true block of motor neurons. *Significance.* Our findings show that kHz stimulation using LIFEs can be precisely controlled to achieve selective conduction block. By leveraging existing knowledge from conventional stimulation techniques, this approach allows for the development of stimulation protocols that effectively block abnormal neural patterns with reduced side effects.

## 1. Introduction

Several neurological disorders lead to neuronal hyperactivity, resulting in undesired muscle and nociception responses, and/or autonomic disturbances [1–4]. Reducing or blocking this neural hyperactivity can alleviate symptoms, but current methods, such as pharmacological or surgical treatments, often suffer from non-specificity, nerve damage, low response times, and limited reversibility [5–7]. In contrast, kilohertz electrical stimulation (KES) applied to the peripheral nervous system offers a rapid, local, and reversible block of action potential conduction [8].

In many cases, the therapy needs to target specific fibers while sparing the rest of the nerve. For instance, the vagus nerve innervates many different organs and limbs [9, 10]. A complete or indiscriminate block of the vagus nerve could disrupt essential functions or cause severe side effects [11]. Patel and Butera highlighted the importance of achieving a partial and selective KES neural conduction block, stressing its necessity for effective clinical application [12].

The spatial selectivity of a neural interface characterizes its ability to access a spatially localized population of fibers without affecting neighboring populations [13]. While spatial selectivity has been extensively studied for neural activation with various electrode designs [14–16] it has been poorly explored for KES neural conduction block. The large majority of *in vivo* conduction block experiments have been performed with extrafascicular electrodes [17], mainly with multi-contact cuff-like electrodes wrapping around the nerve [18]. Extraneural electrodes avoid disruption or damage to the nerve while being relatively simple to implant, but have a limited selectivity.

On the other hand, intrafascicular electrodes such as longitudinal intrafascicular electrodes (LIFEs) [19] have proven to be capable of selective activation of a small population of fibers within a fascicle [19–22], as well as beeing a mechanicaly safe and a stable interface [23]. Biocompatibility has also been demonstrated for such electodes. Implantation within fascicles over a 6 month period resulted in minimal tissue damage, no signs of an inflammatory response, and stable neural recordings, suggesting a well-preserved nerve-tissue interface [22, 24–26].

Although neuronal activation and KES conduction block are two fundamentally different mechanisms [27, 28], certain correspondences can be made. The block threshold, equivalent to the activation threshold, is used to assess the minimum KES amplitude required to block neural activity [29]. Similar to activation threshold, the block threshold increases with the fiber-to-electrode distance, and decreases with fiber diameter: a small unmyelinated fiber requires a higher stimulus to be blocked than a large myelinated [30–32]. A linear frequency-amplitude relationship has been reported in many *in vivo* experiments [33, 34] and *in-silico* modeling [35, 36]. Taking the parallel with neural activation a step further, studying the recruitment curve of the KES block allows us to assess several properties, such as its controllability and selectivity, and therefore makes it possible to quantitatively evaluate the effect of stimulation parameters (e.g., electrode design, frequency, waveform, etc) on the nerve conduction block.

In this work, the ability to induce and control for fascicle-selective neural conduction block with KES using LIFEs is evaluated in the sciatic neve of rodents. In particular, this work tests the hypothesis that LIFEs can produce a controllable KES neural conduction block with minimal interfascicular spillover, and thus a high degree of interfascicular selectivity. In addition, the proximity of LIFE to the fibers suggests lower block thresholds than those obtained with cuff-like electrodes.

To test theses hypotheses, a LIFE used for activation (*aLIFE*) was placed proximally in the targeted fascicle, and a LIFE used for KES blocking (*bLIFE*) was placed distally. Block thresholds and recruitment curves were derived from isometric force recruitement to assess the neural block. KES block selectivity derived from interfascicular spillover was evaluated with *aLIFEs* and *bLIFEs* implanted in two different fascicles. The onset response, the brief unwanted and asynchronous neural activity observed when the KES is turned on [12, 37, 38], was also evaluated as electrode geometry plays a key role in the onset response duration [39]. In addition, the onset response spillover, i.e. the onset response produced in the non-targeted fascicle was also evaluated. Finally, the muscle fatigue caused by the KES block with LIFEs was evaluated and compared to fatigue obtained under conventionnal stimulation for activation.

## 2. Material and methods

*In vivo* acute, non-survival experiments were carried out on eleven (table 1) adult rodents Sprague Dawley, male, weight 400–449 g, median: 428 g). All procedures were approved by the Institute for Animal Care and Use Committee of the University of Arkansas. Ten additional animals were used to finalize and validate the experimental setup and stimulation protocols, which included electrode implantation procedures. No data produced during these development experiments are presented here.

**Table 1.** Summary of the number of LIFEs implanted, the number aLIFE-bLIFE pairs used in each rat, details of the fascicles implanted with each LIFE, and the number and specifics of the datasets collected per rat. B_TH_ datasets were collected using the KES Block Threshold protocol SEL datasets were collected using the KES Block Selectivity and Recruitment Curves protocol ONSET datasets were obtained using the KES Onset Response protocol and FATIGUE datasets were acquired using the Muscle Fatigue Induced by Control Scenarios protocol. For further details, see section 1.2 and table 1–4 in appendix 2 (supplementary data).

			# and location of implanted LIFEs			# Dataset collected			
Animal ID	Total LIFE	# of aLIFE-bLIFE pairs	*aLIFE* in TF	*aLIFE* in PF	*bLIFE* in TF	*B*_TH_	SEL	ONSET	FATIGUE
RAT1	3	2	2	—	1	2	2	1	*—*
RAT2	3	2	2	—	1	2	2	1	*—*
RAT3	4	3	1	—	3	3	2	2	*—*
RAT4	3	2	1	1	1	1	2	1	*—*
RAT5	3	2	1	—	2	2	—	1	*—*
RAT6	2	2	2	—	—	—	—	1	2
RAT7	4	3	3	—	1	—	—	—	3
RAT8	2	2	2	—	—	—	—	—	2
RAT9	3	2	—	2	1	—	2	1	*—*
RAT10	5	6	—	2	3	—	5	6	*—*
RAT11	5	6	—	2	3	*—*	4	6	*—*

### 2.1. Material

#### 2.1.1. Activation and KES block electrodes

Custom-fabricated monopolar LIFEs with a single exposed contact were used for both the neural activating stimulation (*aLIFEs*) and the neural conduction block via KES delivery (*bLIFEs*). LIFEs were made using a thin wire made of platinum/iridium (Pt/Ir) and a diameter of 27.5 *μ*m. A 75 *μ*m tungsten needle was attached to one end of the wire and served as a guide during implantation. A metal plate was soldered to the other end of the wire and was used to connect to the neurostimulator.

The total length of the electrode was about 30 cm. An active site was exposed approximately 1 cm away from the tungsten needle connection by removing the wire insulation with a coating thermal removal technique. During the preliminary investigation, the KES block was obtained more readily when the *bLIFE* active site was larger than that of the *aLIFE*. Therefore, we made the electrode pairs according to this principle. The impedance of each electrode was measured and used to ensure manufacturing consistency (appendix 1 in supplementary data). Impedance at 1 kHz was 28.0 kΩ (std: 12.5 kΩ) for the stimulation electrodes and 8.9 kΩ (std: 2.3 kΩ) for the blocking electrodes. LIFEs’ active-site length was calculated from impedance measurement to about 0.6 mm (std: 0.3 mm) for the *aLIFEs* and about 1.2 mm (std: 0.4 mm) for the *bLIFEs* [41].

#### 2.1.2. Electrophysiological setup

A custom neurostimulator presented in [40] was used to perform both the stimulation and the KES neural conduction block. The neurostimulator is capable of outputting arbitrary waveforms on 8 independent channels, with a maximum current of 1 mA per channel. Channels can be ganged to increase the maximum current delivered on a given electrode. High-frequency capabilities of the stimulator using LIFEs were demonstrated in [40]. A DC-blocking circuit adapted from [37] was added between the electrodes and the stimulator outputs to avoid any DC contamination [42]. Component values were adjusted to accommodate the higher impedance of the LIFEs.

Isometric force recordings were conducted with a 6-axis force transducer (JR3 20E, JR3 Inc., USA). The paw of the rodent was secured to the force transducer via a 3D-printed custom fixture. The baseline of each axis of the force transducer was adjusted before the experiment using on-board trimmers. Outputs of the force transducer were sampled at 30 kS s^−1^ with a dedicated data acquisition system (Scout processor, Ripple Neuro, USA). Acquisition and stimulation were synchronized using the integrated triggers of both systems and custom Python scripts.

#### 2.1.3. Animal preparation

Animals were maintained under anesthesia with Isoflurane (1.5%–3%) during the experiment, typically around 6–7 h. Surgical depth was assessed using the toe pinch reflex and continuous monitoring of the rodent’s vital signs. Isoflurane concentration was adjusted accordingly. Rat body temperature was maintained between 35 °C–38 °C with a heated blanket and monitored using a rectal temperature probe (MicroTherma ThermoWorks Inc., USA). Heart rate and blood oxygenation were monitored using pulse oximetry (SurgiVet V9203, Smiths Medical ASD Inc., USA). Saline solution was injected subcutaneously at 2cc h^−1^.

The rat was grounded with a needle inserted into the skin on the back of the animal. The needle also served as a current return electrode for the electrical stimulation.

The left hindlimb was shaved, and a first incision was made on the thigh. The muscles were retracted, the nerve isolated from the surrounding connective tissue, and an epineural dissection used to expose the fascicles and visualize the bands of Fontana. A 3D-printed custom tool was used to lift the nerve and facilitate the implantation of the electrodes [43]. LIFEs were threaded parallel to the axons into the target fascicle (tibial fascicle—TF, or common peroneal fascicle—PF), approximately 1.5–2 cm proximal to the bifurcation of the tibial and common peroneal nerves. A 100 *µ*s, 50 *µ*A biphasic square pulse with a pulse frequency of 1 Hz was applied to each electrode individually. The electrode was then gently pulled back until a visible muscle twitch was observed, confirming that the LIFE’s active site was correctly positioned within the fascicle. The LIFEs were then securely fastened to the epineurium using 8-0 non-absorbable sutures. Finally, the muscle layers and skin were closed with 5-0 non-absorbable sutures.

**Figure 1.**
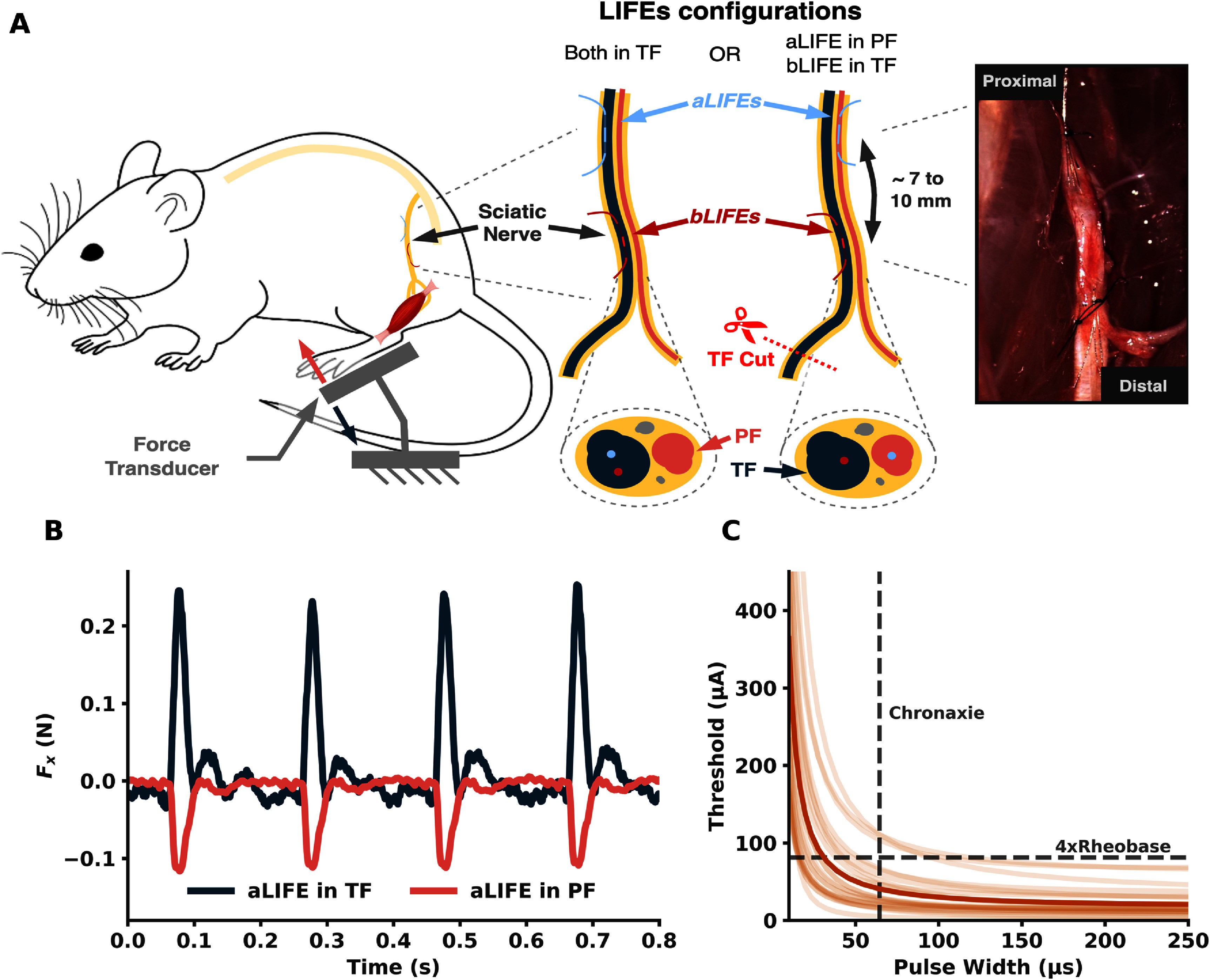
*In-vivo* experiment setup to demonstrate KES neural conduction block with LIFEs. (a) LIFEs were implanted in the sciatic nerve of anesthetized rats for both neural activation (aLIFEs) and KES neural conduction block (*bLIFEs*). *bLIFEs* are implanted in the tibial fascicle (TF), while aLIFEs are implanted in TF or in the peroneal fascicle (PF). The paw of the animal was secured to a 3D-printed fixture and attached to the force transducer. LIFEs are connected to a custom neurostimulator [40] via a DC-blocking circuit (not shown). (b) Measured force along the *x*-axis of the force transducer with two aLIFEs implanted in different fascicles. A positive force represents plantarflexion of the paw, indicating that the stimulating electrode is located inside TF. A negative force represents dorsiflexion, indicating that the stimulating electrode is in PF. (c) Strength-Duration (SD) curves of the implanted electrode (*N*_LIFEs_ = 24). Darker trace is the mean SD curve. Rheobase and chronaxie values of each curve were used to define the stimulation pulse parameter of the aLIFEs.

For each rat, 1–3 *bLIFEs* were implanted distally in the TF, and 1–3 *aLIFEs* were implanted proximally, either in the TF or the PF. The distance between them was ∼ 0.7–1 cm (figure 1(a)). The number of *aLIFEs* and *bLIFEs* implanted per rat, along with their implantation sites (TF or PF), are summarized in table 1. The use of a third distal activating LIFE to assess the status of the neuromuscular junction during KES delivery, as done in [34], was not feasible in this study due to the limited length of the exposed nerve.

At the end of the experiment, rats were euthanized with an overdose of isoflurane, the exposure was maintained for more than 1 min after the rat stopped breathing.

### 2.2. Stimulation protocols

#### 2.2.1. Electrode location and strength-duration (SD)curve

A 100 *μ*s biphasic square pulse with a pulse frequency of 5 Hz was applied to each implanted *aLIFE* and *bLIFE*, one by one, to evoke muscle twitches.

The induced rotation of the ankle (plantarflexion or dorsiflexion) was used to assess the fascicle in which the electrode was implanted (figure 1(b)). Plantarflexion indicates activation of the plantar flexor muscles (gastrocnemius and soleus), that are innervated by the TF. Dorsiflexion indicates activation of the dorsiflexor muscles (anterior tibialis and peroneus longus) and are innervated by the PF of the sciatic nerve [44].

Simultaneous neural activation in TF and PF can lead to co-contraction, which may distort the force analysis. However, it has been shown that stimulation with LIFEs can elicit neural responses only within the implanted nerve fascicle [45]. Studies conducted by our group confirmed these results with our experimental setup (unpublished data see appendix 4 in the supplementary data). As an additional safety measure, we set the stimulation amplitude just above the threshold that induces a visible muscle response, thereby ensuring that only the motor axon pool closest to the electrode is activated.

If co-contraction occurred, the electrode was deemed unsuitable and not used, as it indicated improper implantation across both the TF and PF fascicles.

SD curves were evaluated for each *aLIFE* and *bLIFE* electrode (figure 1(c) and table 1)). Cathodic duration (PW) was swept from 10 *μ*s to 250 *μ*s with a 1:1 cathodic-anodic ratio. Pulse repetition was set to 1 Hz. The stimulation amplitude was gradually increased until a visible twitch was observed, which defined the activation threshold (*A*_TH_). Electrodes were discarded when the SD curve could not be obtained (e.g., muscle twitches were no longer observed when delivering the stimulus). SD curves are later used to define stimulation parameters of the *aLIFE*.

#### 2.2.2. KES stimulation scheme

**Figure 2.**
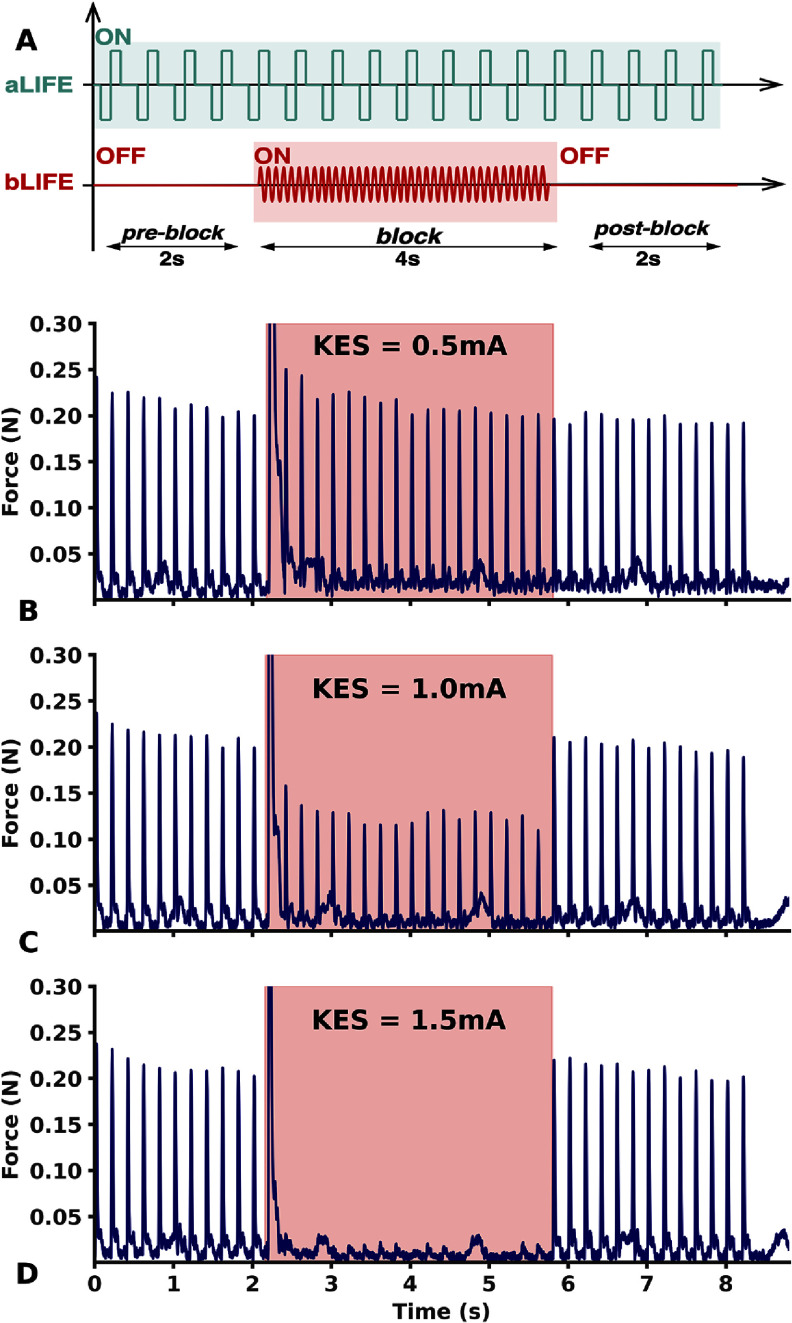
KES Block stimulation scheme. (a) Current stimuli applied to the *aLIFE* and *bLIFE* to demonstrate and characterize KES neural conduction block with LIFEs. The subthreshold conventional stimulus was applied throughout the trial KES was applied only during the block phase. (b)–(d): Examples of neural block observed with LIFEs and KES. KES frequency was set to 15 kHz and KES amplitude at (b) 0.5 mA (c) 1.0 mA (d) 1.5 mA.

The KES stimulation scheme consisted of a cycle with three distinct phases (figure 2(a)): a ‘pre-block’ phase, where only proximal neural activation was delivered via the *aLIFE* for approximately 2 s, followed by a ‘block’ phase that combined proximal neural activation via the *aLIFE* with distal KES block delivered through the *bLIFE.* Preliminary studies showed that the onset response during KES delivery was brief, typically under 1 s, and we did not observe any long-lasting onset response. Based on this observation, we chose a block-phase duration of 4 s. Finally, the ‘post-block’ phase involved only proximal neural activation via the *aLIFE* for approximately 2 s.

The pre- and post-block phases ensured that the *aLIFE*-induced neural activation was effective before and after the delivery of the KES block via the *bLIFE*. A minimum rest period of 20 s was imposed between each cycle to reduce neural and muscular fatigue caused by the electrical stimulation.

We derived specific protocols from this general scheme to investigate particular aspects of the KES response, such as the KES block threshold (*B*_TH_), the KES block selectivity, the onset response, and the muscle fatigue. These protocols are described in detail below. Each protocol could be performed in the same rat with different *aLIFE-bLIFE* combinations. The details of the protocols performed are described in the table 1.

For each specific protocol, the *aLIFE*’s proximal neural activation used a 5 Hz supra-threshold biphasic square pulse. The pulse width was set to chronaxie value (mean: 64 *µ*s, std: 26 *µ*s), and the pulse amplitude (unless otherwise specified) was set to 4 times the rheobase value (mean: 81 *µ*A, std: 71 *µ*A) measured with the *aLIFE*’s SD curve (figure 1(c)).

The KES was delivered distally by the *bLIFE* in the form of a symmetrical sine wave with an initial phase of 0°. The amplitude and frequency of the KES were adjusted according to the specific protocol investigated.

#### 2.2.3. KES block threshold

The KES amplitude was gradually increased between each cycle of the KES block scheme until a visible reduction in the force produced was observed during the block phase (figures 2(b) and (c)). The blocking threshold (*B*_TH_) for the *aLIFE* and *bLIFE* combination was identified by increasing the KES amplitude until the peak-force produced was less than 5% than the peak-force measured during pre-block phase.

The *B*_TH_ was measured with KES frequencies from 5 kHz to 30 kHz, with a 5 kHz increment, a frequency range commonly used in the literature [18, 31, 32].

The collection of *B*_TH_ values measured across the KES frequency range for an *aLIFE-bLIFE* pair constitutes a *B*_TH_ dataset. A total of 10 *B*_TH_ datasets were collected, corresponding to 10 combinations of *aLIFE-bLIFE* par in TF, distributed across 5 rats (RAT1 to 5). All experimental details and stimulation parameters can be found in table 1 of the appendix 2 (supplementary data).

#### 2.2.4. KES block selectivity and recruitment curves

We conducted a KES amplitude sweep to characterize its effect on the force measured during the block phase. Two distinct scenarios were examined: when both the *aLIFE* and *bLIFE* are implanted in TF, and when the *bLIFE* is implanted in TF while the *aLIFE* is implanted in PF (figure 1(a)).

The first scenario aims to construct the recruitment curve of the KES block in TF, while the second evaluates KES block spillover in PF. Data from both scenarios will be used to assess the interfascicular selectivity of the KES block with LIFEs.

In the first scenario, KES amplitude was increased from 0.5*B*_TH_ to 1.5*B*_TH_ in steps of 0.05*B*_TH_. In the second scenario, KES amplitude was increased from 0.5*B*_avg_ to 1.5*B*_avg_ in steps of 0.05*B*_avg_, where *B*_avg_ is the average value of the block thresholds measured per frequency.

In this second scenario, the onset response induced by KES delivery in TF generates plantarflexion, which mechanically interferes with the dorsiflexion produced by the *aLIFE* in PF. Although this co-contraction lasts less than 1 s, preliminary studies have shown that it leads to a lasting reduction in force produced by the dorsiflexor muscles, likely due to muscle fatigue induced by the co-contraction.

To prevent distortion in the force readout, onset co-contractions were inhibited in this second scenario by cutting the tibial nerve branch distal to its bifurcation (figure 1(a)).

For each scenario, amplitude sweeps were done with KES frequencies of 10 kHz, 15 kHz, and 20 kHz. The aggregation of amplitude sweeps conducted at these three frequencies for a pair of *aLIFE-bLIFE* constitutes a SEL dataset. We collected a total of 7 datasets (RAT1 to 4) with both *aLIFE* and *bLIFE* implanted in TF, and 11 datasets (RAT4 and 9–11) with *bLIFE* in TF and *aLIFE* in PF (and with the tibial nerve branch cut). All experimental details and stimulation parameters are provided in Table 3 of Appendix 2 (Supplementary Data).

#### 2.2.5. KES onset response

We characterized the onset response produced during KES delivery through the *bLIFE* (implanted in the TF) as a function of KES amplitude, for KES frequencies of 10 kHz, 15 kHz, and 20 kHz. The *aLIFE* stimulation amplitude was kept at 0 *µ*A throughout the protocol to isolate the effect of the KES onset on the force readout.

The aggregation of amplitude sweeps conducted at these three frequencies for one *bLIFE* constitutes a single ONSET dataset. We collected a total of 19 ONSET datasets across 9 different rats. Detailed information on the experiments and stimulation parameters can be found in table 4 of appendix 2 (supplementary data).

The first 13 datasets (ONSET0 to ONSET12) were collected with the tibial nerve branch intact, allowing us to primarily observe the force generated by the muscles innervated by the TF. The remaining 7 datasets (ONSET13 to ONSET19) were collected with the tibial nerve branch cut (figure 1(a)) enabling us to observe the force produced by the muscles innervated by the PF and thus to characterize the onset spillover between the TF and PF.

#### 2.2.6. Muscle fatigue induced by control scenarios

We evaluated the muscle fatigue caused by the KES stimulation protocol and compare it to fatigue resulting from two control scenarios (figure 4 in appendix 5, supplementary data).

The first scenario, ‘no-KES,’ evaluates muscle fatigue caused solely by *aLIFE* neural activation. In this scenario, the KES amplitude of *bLIFE* was set to 0 *µ*A throughout, including during the block phase (figures 4(a) and (b) in appendix 5, supplementary data).

The second fatigue control scenario is designed to simulate an ideal neural conduction block that would not induce any additional muscle fatigue besides that caused by *aLIFE* stimulation alone, and would allow the muscle to recover during the block phase as it remains unstimulated. This scenario is referred to as the ‘*ideal-block*’, and it involved the KES block scheme with both *aLIFE* and *bLIFE* stimulations turned off during the block phase figures 4(c) and (d) in appendix 5, supplementary data).

For both control scenarios, the protocol was repeated 10 times, with a 20-second rest period between repetitions. The set of 10 repetitions for each scenario (‘*no-KES*’ and ‘*ideal-block*’) constitutes a single FATIGUE dataset. A total of 7 FATIGUE datasets were collected across 3 different rats (RAT 6–8). Detailed information of the experiment can be found in table 2 of appendix 2, in the supplementary data.

### 2.3. Data analysis

Data post-processing, analysis and visualization were done using code developed in the Python 3 language, using the NumPy, SciPy, Statsmodels, Pandas, Matplotlib and Seaborn external librairies.

#### 2.3.1. KES block threshold

Block thresholds are displayed as a function of the KES frequency [18, 31, 32]. Additionally, we plotted them as a function of the KES half-period *T*_KES_ defined as follows:

\begin{equation*}{T_{{\text{KES}}}} = \frac{1}{{2{f_{{\text{KES}}}}}}\end{equation*}

where *f*_KES_ is the KES frequency. With these values a block-duration curve (BD) was plotted, analogous to the SD activation curve.

The injected charge density at the block threshold *B*_TH_ was evaluated:

\begin{equation*}{ }{\sigma _{{\text{per phase}}}} = \frac{{{Q_{{\text{per phase}}}}}}{{{S_{{\text{LIFE}}}}}}\end{equation*}

where [46]:

\begin{equation*}{ }{Q_{{\text{per phase}}}} = \frac{{2{B_{{\text{TH}}}}{T_{{\text{KES}}}}}}{\pi }\end{equation*}

and:

\begin{equation*}{ }{S_{{\text{LIFE}}}} = {{\unicode{x00F8}}_{{\text{LIFE}}}}.{L_{{\text{LIFE}}}}.\pi \end{equation*}

where ${{\unicode{x00F8}}_{{\text{LIFE}}}}$ and ${L_{{\text{LIFE}}}}$ are the diameter and the active-site length of the *bLIFE*.

#### 2.3.2. KES block protocol

Force baseline was evaluated during a 1 s blank recording prior to each block protocol cycle. Recorded data were adjusted accordingly and digitally high-pass and low-pass filtered (resp. 0.1 Hz and 100 Hz).

Using the stimulator triggers, the recording is divided into three sections corresponding to the three phases of the block protocol. For each phase, the stimulus-triggered force average was calculated to obtain a single twitch. The stimulus-triggered averaged twitch was evaluated from 10 twitches for the *pre*- and post-*block* phases. 40 twitches are produced during the *block* phase, but the first 10 are discarded to eliminate the effect of the onset response.

For each stimulation phase, we evaluate the stimulus-triggered twitch average peak-force and area under the curve (AUC). The overall procedure is schematized in figure 5 of appendix 6, supplementary data.

#### 2.3.3. KES interfascicular selectivity

We define the amount of KES block as follow:

\begin{equation*}{\text{BLK}}\left( {{I_{{\text{KES}}}},{f_{{\text{KES}}}}} \right) = 1 - \frac{{{X_{{\text{block}}}}\left( {{I_{{\text{KES}},{ }{f_{{\text{KES}}}}}}} \right)}}{{{X_{{\text{pre}}}}}}\end{equation*}

where ${X_{{\text{phase}}}}{ }$ represents either the peak force or AUC during the block and pre-block phases from the SEL datasets, and ${I_{{\text{KES}}}}$ denotes the KES amplitude.

We assessed interfascicular selectivity by deriving a selectivity index, *S_i_*, previously used to assess interfascicular selectivity of neural activation [45, 47, 48], and we adapted it to KES conduction block:

\begin{equation*}{ }{S_i} = {\text{BL}}{{\text{K}}_i} - \frac{1}{{N - 1}}\sum\limits_{j \ne i} {\text{BL}}{{\text{K}}_j}\end{equation*}

where *i* is the fascicle of interest and *N* is the number of fascicles considered.

Here we consider only PF and TF, so (7) is simplified to:

\begin{equation*}{ }{S_{{\text{TF}}}} = {\text{BL}}{{\text{K}}_{{\text{TF}}}} - {\text{BL}}{{\text{K}}_{{\text{PF}}}}\end{equation*}

where ${\text{BL}}{{\text{K}}_{{\text{TF}}}}$ and ${\text{BL}}{{\text{K}}_{{\text{PF}}}}$ are the amount of KES block measured in the TF (datasets SEL0 to 6 in Table 3 of Appendix 2, Supplementary Data) and PF respectively (datasets SEL7 to 18).

#### 2.3.4. KES recruitment curves

We fitted the amount of block in TF (datasets SEL0 to 6 in table 3 of appendix 2, supplementary data) versus the KES amplitude with the following sigmoid function using nonlinear least squares:

\begin{equation*}{\text{ BLK}}\left( {{I_{{\text{KES}}}}} \right) = \frac{1}{{1 + {{\text{e}}^{ - \beta \left( {{I_{{\text{KES}}}} - \sigma } \right)}}}}\end{equation*}

where $\sigma $ represents the KES amplitude required to reach 50% of block and $\beta $ is related to the KES block recruitment rate (i.e., the slope of the sigmoid function’s linear region). We defined the fitting boundaries to ensure that the resulting *σ* and *β* parameters were not confined to either extreme ($0.5 &lt; \sigma &lt; 3\,{\text{mA}};3 &lt; \beta &lt; 30\,{\text{m}}{{\text{A}}^{ - 1}})$.

#### 2.3.5. KES onset-response

We characterized the KES-induced onset response by its peak-force amplitude, AUC and time duration (TD). We defined TD as:

\begin{equation*}{\text{ TD}} = {t_{0.1f}} - {t_{0.1i}}\end{equation*}

where ${t_{0.1i}}$ and ${t_{0.1f}}$ are respectively the initial and final time at which the onset-response is egal to 10% of its peak-value value.

We distinguished the onset response produced by motor fibers in TF (datasets ONSET0 to 12 in table 4 of appendix 2, Supplementary Data) from the onset spillover generated by motor fibers in PF (datasets ONSET 13–19 datasets).

Peak-force, AUC and TD values were normalized to the maximum value measured in each ONSET dataset. Un-normalized onset response plots are available in figure 9 of appendix 10 (supplementary data).

#### 2.3.6. Evaluation of muscle fatigue

Muscle fatigue caused by the KES protocol was evaluated by comparing the force profiles during the pre-block and post-block phases. The fatigue ${f_X}$ was defined as:

\begin{equation*}{f_X} = 1 - { }\frac{{\left| {{X_{{\text{pre}}}} - {X_{{\text{post}}}}} \right|}}{{{X_{{\text{pre}}}}}}{\text{ }}\end{equation*}

where ${X_{{\text{pre}}}}$ and ${X_{{\text{post}}}}$ are a twitch characteristic (peak-force or AUC) measured during the *pre-block* phase and the *post-block* phase, respectively.

We evaluated the muscle fatigue from the two control scenarios ‘*no-KES*’ and ‘*ideal-block*’ (FATIGUE0 to 6 datasets, in table 2 of appendix 2, supplementary data).

We also evaluated muscle fatigue during KES block using datasets SEL0 to SEL6. For these datasets, we created two distinct groups: one assessing muscle fatigue when the KES amplitude produced a block greater than 0% but less than 25% (*weak-block* group), and another evaluating muscle fatigue when the KES block was greater than 75% (*strong-block* group).

The *weak-block* and *strong-block* groups will validate that stronger KES neural block of motor fibers results in less muscle fatigue, as the muscle is less recruited during the block phase and therefore has a greater opportunity to recover from fatigue.

## 3. Results

### 3.1. KES block thresholds

**Figure 3.**
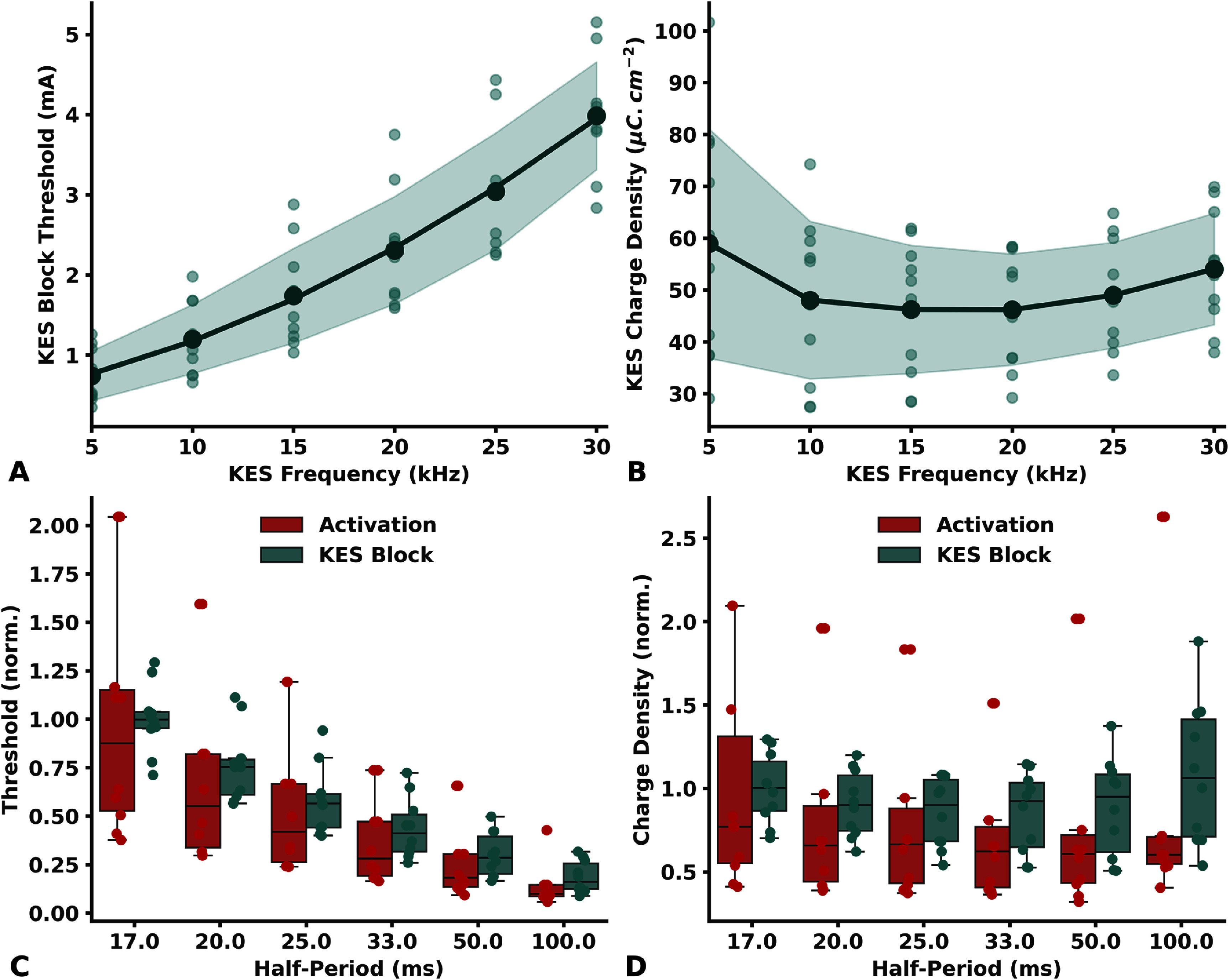
KES block threshold with LIFEs. (a) *B*_TH_ and (b) corresponding charge density versus KES frequency. Larger and darker markers represent the mean *B*_TH_ (resp. charge density) evaluated for each KES frequency. The shaded area corresponds to the mean ± std deviation zone. (c) and (d) present the normalized threshold and charge density boxplots for neural activation (in red) and KES conduction block (in green) threshold and charge density are plotted versus the half-period stimulus, which corresponds to the PW of the biphasic stimulus and the sinusoidal half-period of the KES stimulation for neural activation and KES, respectively. Thresholds and charge density were normalized to their mean value obtained with a 17us half-period stimulus. Non-normalized activation thresholds are depicted in figure 6 of appendix 7 (supplementary data).

KES block thresholds versus KES frequency are shown in figure 3(a). For two animals, block threshold with a 5 kHz KES could not be measured due to a long-lasting onset response. KES block was obtained in all animals tested between 10 kHz and 30 kHz, and was measured between 0.35 mA with a 5 kHz KES and up to 5.2 mA with a 30 kHz KES. In the tested frequency range, block thresholds were monotically increasing with KES frequency for every *aLIFE-bLIFE* pair, from 0.74 mA (std = 0.31 mA) with a 5 kHz KES frequency to 3.98 mA (std = 0.67 mA) with a 30 kHz KES frequency.

The linear regression between KES frequency and *B*_TH_ applied to each *B*_TH_ dataset resulted in a mean *R*^2^ of 0.97 (min = 0.89, max = 0.99, std = 0.03). We also applied a quadratic regression to the data, which improved the fit, yielding a mean *R*^2^ > 0.99 (min = 0.99, max > 0.999, std = 0.003) with a small but significant quadratic term (*p* = 0.006).

Assuming a monotonic relationship, if one exists, between *bLIFE* length and *B*_TH_, we calculated the Spearman rank correlation coefficient between the quadratic regression coefficients and the length of the *bLIFE* active site. No significant correlation was observed (*p* > 0.43, *p* > 0.60, and *p* > 0.59). Additionally, no significant Spearman correlation was found between the *bLIFE* active site length and the thresholds measured at 10 kHz, 20 kHz, and 30 kHz (*p* > 0.78, *p* > 0.76, and *p* > 0.52, respectively).

The injected charge density per phase versus KES frequency exhibits a non-monotonic relationship with a minimum of $46\mu {\text{C}} \cdot {\text{c}}{{\text{m}}^{ - 2}}$ (std = $10.7\mu {\text{C}} \cdot {\text{c}}{{\text{m}}^{ - 2}}$) obtained with a 20 kHz KES frequency (figure 3(b)).

The shape of the KES block threshold and the injected charge density with *T*_KES_ is very similar to the shape of the activation threshold with PW. (figures 3(c) and (d)). Indeed, there were no significant differences between the normalized SD and BD curves and the charge density per phase curves (unpaired *t*-test, *p* > 0.59 and *p* > 0.53 respectively). However, the minimum injected charge density per phase was obtained at *T*_KES_ = 25 *µ*s (*f*_KES_ = 20 kHz) for the KES block and around PW = 33 *µ*s for the activation threshold.

### 3.2. Interfascicular selectivity

**Figure 4.**
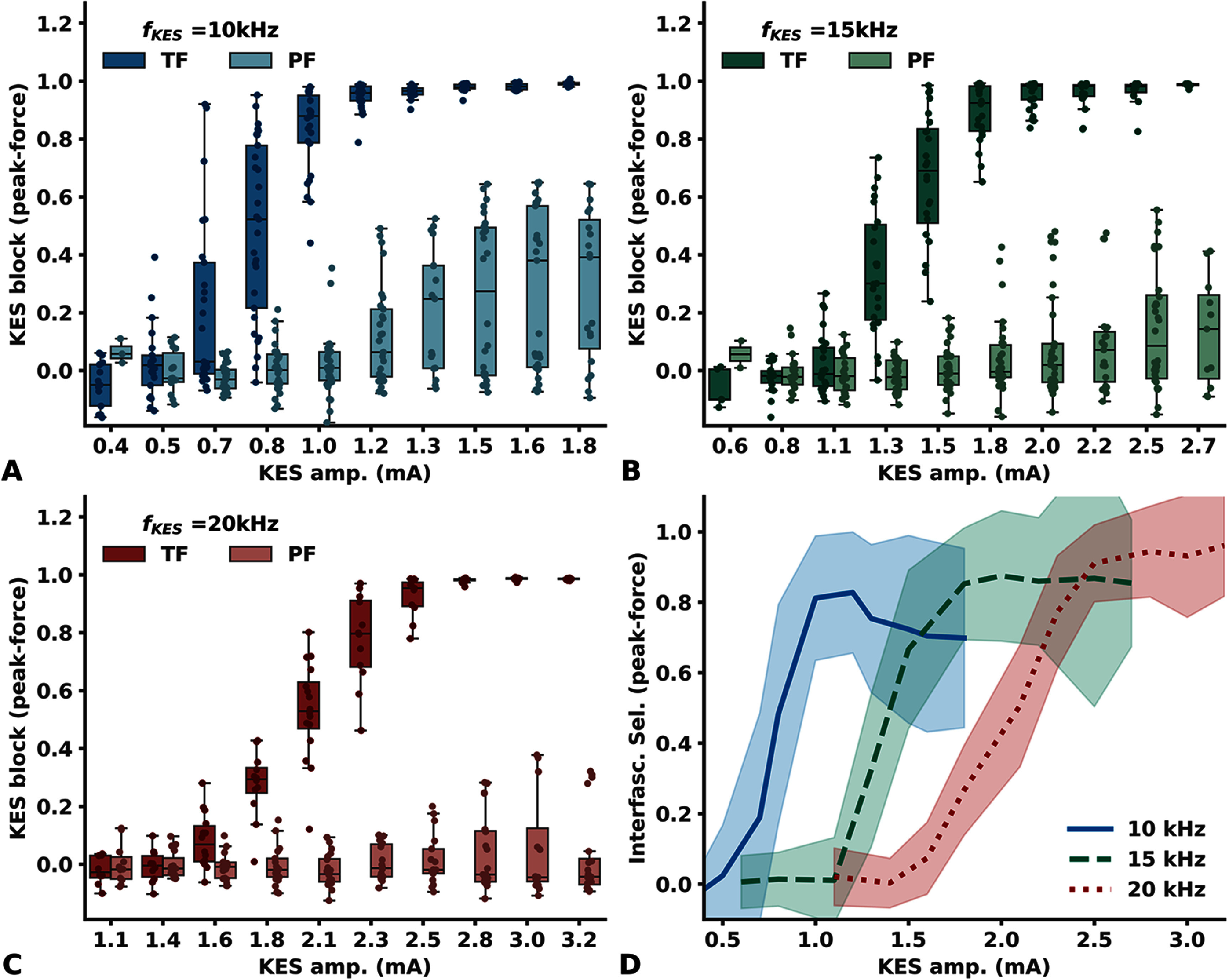
KES Block Interfascicular Selectivity with LIFEs. KES block measured from twitch peak-force with a KES frequency of (a) 10 kHz (b) 15 kHz (c) 20 kHz. Darker boxes represent block in TF lighter boxes represent block in PF (d) Resulting interfascicular selectivity index with the 10 kHz frequency (blue) 15 kHz frequency (green) and 20 kHz frequency (red). Each line and filled area correspond to the mean ± std deviation zone for that frequency.

The amount of KES block, derived from the twitch peak-force, as a function of KES amplitude is shown in figure 4(a) for KES frequencies of 10 kHz, (b) 15 kHz and (c) 20 kHz.

When *aLIFE* and *bLIFE* are implanted in the TF, a progressive increase in KES amplitude leads to a progressive increase of the conduction block, reaching 100% of peak-force block for the three tested frequencies.

When the *bLIFE* is in TF but the *aLIFE* is activating motor fibers in PF, none of the tested combinations of KES amplitude and frequency resulted in a 100% block of PF. This was observed with a KES frequency of 10 kHz and a KES current up to 1.8 mA, 15 kHz and a KES current up to 2.7 mA and 20 kHz with a KES current up to 3.2 mA. Maximum block was about 29% (std = 25%), 13% (std = 18%) and 5% (std = 14%) with a 10 kHz, 15 kHz, and 20 kHz KES frequency respectively.

At KES amplitudes of 1.2 mA, 1.8 mA, and 2.5 mA, corresponding to *B*_TH_ at 10 kHz, 15 kHz, and 20 kHz KES frequencies, respectively, KES block was greater than 90% in TF, but was 12% (std = 17%) in PF with a 10 kHz frequency and insignificant at 15 kHz and 20 kHz frequency. It should also be noted that in 3 datasets (2 different rats) we observed no block in the PF over the entire KES amplitude range tested and for all 3 frequencies tested.

KES block selectivity indices versus KES amplitude, for a frequency of 10 kHz, 15 kHz and 20 kHz, are shown in figure 4(d). The selectivity peaks at approximately 0.83 (std = 0.17) at 10 kHz, 0.88 (std = 0.18) at 15 kHz, and 0.95 (std = 0.14) at 20 kHz. For a 10 kHz KES frequency, this maximum selectivity was observed for a KES amplitude just below *B*_TH_, and then decreased due to interfascicular KES block spillover to 0.70 (std = 0.25) at the maximum KES amplitude tested (1.8 mA). Similar results were observed with a 15 kHz KES frequency, with a less pronounced decrease in selectivity down to 0.85 (std = 0.18) with a 2.7 mA KES block. The maximum KES amplitude tested does not show a decrease in interfascicular selectivity when the KES frequency is 20 kHz.

Overall, similar results are obtained by analyzing AUC instead of the muscle twitch peak-force (figure 7 in appendix 8, supplementary data).

### 3.3. KES block recruitment curves

**Figure 5.**
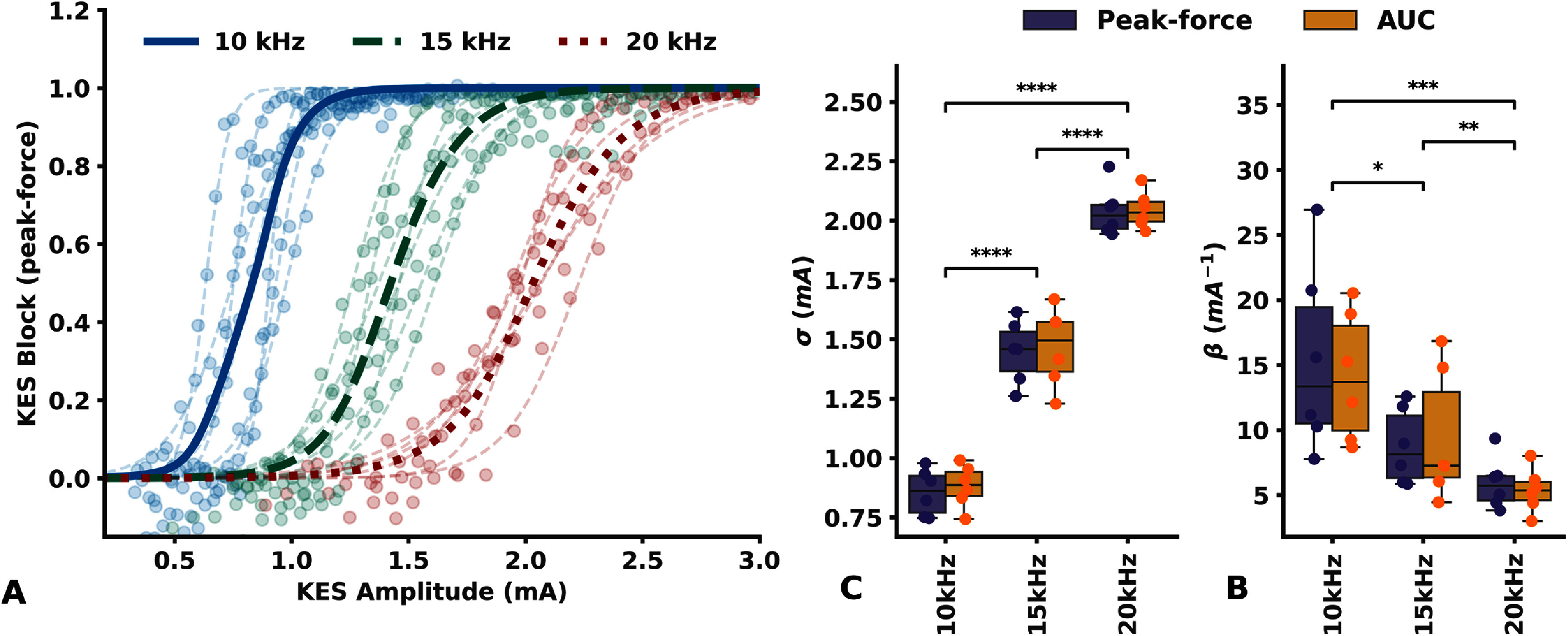
KES Block Recruitment curves. (a) Recruitment curves measured with both aLIFE and bLIFE in TF and derived from twitch peak-force. Blue, green and red lines and markers represent data from 10 kHz, 15 kHz, and 20 kHz KES frequencies, respectively. Each dot is a measured data point each dashed line is the resulting fitted sigmoid function of each dataset Plain thicker lines are the sigmoid function derived from the individual fits mean parameters value (b) Resulting sigmoid fit parameter *σ* and (c) *β* Purple boxes are parameter from recruitment curves fit derived from twitch peak-force yellow boxes are from AUC. Significance is evaluated with a repeated measures ANOVA followed by a Bonferroni Post-hoc Test * *p* < 0.05 ** *p* < 0.01 *** *p* < 0.001 **** *p* < 0.0001 Difference between fit parameters derived from AUC and from peak-force was not significant (*p* = 0.56 and *p* = 0.38, not shown).

Block recruitment curves (*aLIFE* and *bLIFE* are in TF, SEL0 to SEL6 in table 3 of appendix 2, supplementary data) fitted with a sigmoid function are plotted in figure 5(a) for data derived from twitch peak-force and in figure 8 of appendix 9 (supplementary data) for AUC. The fitted function parameters $\sigma $ and $\beta $ are plotted in figures 5(b) and (c).

The relationship of KES block versus amplitude is well explained by the sigmoid function, with an average *R*^2^ of around 0.98 (min = 0.92 max > 0.99 std = 0.02) when peak-force is used for the three frequencies tested. Equivalent results were obtained with recruitment curves obtained from the AUC (mean = 0.96 min = 0.78 max = 0.99, std = 0.05). We also observed no significant difference between the *σ* and *β* parameters obtained from AUC or peak-force (Repeated measures ANOVA, *p* > 0.38 and *p* > 0.56 respectively).

Increasing the KES frequency resulted in a right-shift of the block recruitment curve which translates to a significative increase of the sigmoid fit $\sigma $ parameter (Repeated measures ANOVA, *p* < 0.0001). Increasing the KES frequency also resulted in a significative decrease in the sigmoid fit $\beta $ parameter (Repeated measures ANOVA, *p* = 0.004), and therefore in the KES block recruitment rate.

### 3.4. KES onset-response

**Figure 6.**
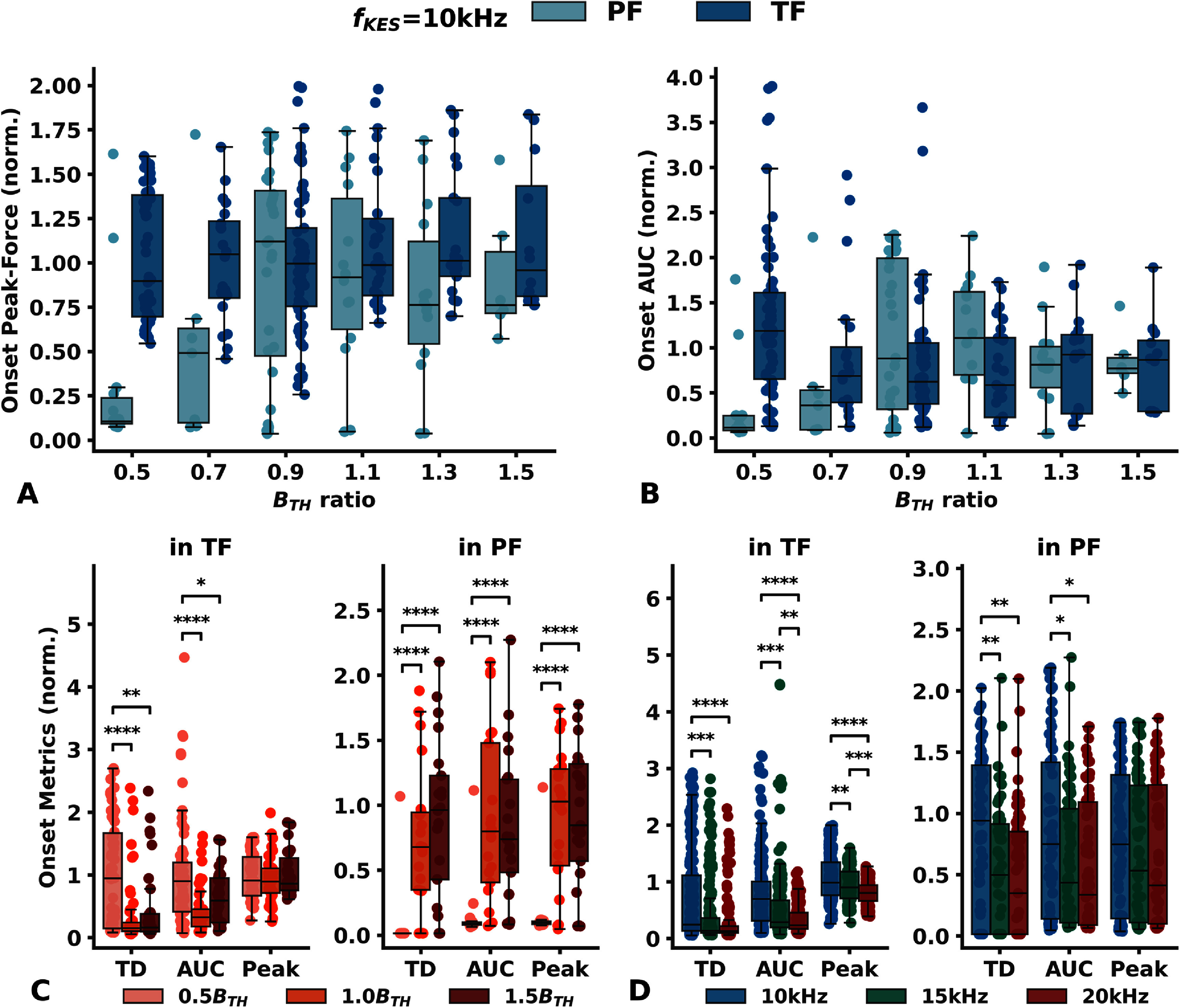
Onset response metrics analysis. (a) Normalized onset peak force and (b) AUC produced by the delivery of a 10 kHz KES signal in TF (dark blue) and PF (light blue) versus KES amplitude. The KES amplitude is expressed as a ratio of the block threshold *B*_TH_ Un-normalized onset response plots are available in figure 9 of appendix 10 (supplementary data) (c) Analysis of the onset in TF (left) and in PF (right) metrics (TD, AUC and peak-force) versus when KES amplitude is about 0.5*B*_TH_ (pink) 1.0*B*_TH_ (orange) 1.5*B*_TH_ (red) (d) when the KES frequency is set to 10 kHz (blue) 15 kHz (green) 20 kHz (red) in TF (left) and in PF (right). Significance is evaluated with a repeated measures ANOVA followed by a Bonferroni Post-hoc Test * *p* < 0.05 ** *p* < 0.01 *** *p* < 0.001 **** *p* < 0.0001.

Onset peak-force and AUC versus KES amplitude with a 10 kHz KES frequency are shown on figures 6(a) and (b). Peak-force, AUC, and TD versus KES amplitude with a 15 kHz and 20 kHz KES are provided in figures 9 and 10 of appendix 10 (supplementary data).

We observed an onset-response in the TF in across all data sets. In 4 ONSET datasets (2 rats) we observed a long lasting onset response (>5 s) when the KES frequency was set to 5 kHz. For KES frequencies of 10 kHz or higher, and for KES amplitudes ranging from 0.5*B*_TH_ to 1.5*B*_TH_, the observed onset response lasted less than 2 s.

We also observed a noticeable onset response in the PF despite delivery of the KES in TF, indicating onset spillover. It is worth noting that in 3 datasets (2 rats), no onset in the PF across the entire KES amplitude range tested, for all 3 KES frequencies. These datasets correspond to those in which no block in PF was observed neither.

While KES amplitude has no clear effect on the onset response peak force in TF, the onset spillover (in PF) increased as the KES amplitude was increased (figures 9 and 10 of appendix 10, supplementary data).

Increasing KES amplitude between 0.5*B*_TH_ and 1.5*B*_TH_ has no significant effect on the onset peak-force in TF (Repeated measures ANOVA, *p* > 0.9), but significantly reduces both the onset AUC and onset TD (*p* < 0.0003 for both metrics). Specifically, increasing the KES amplitude from 0.5B *B*_TH_ to 1.0*B*_TH_ significantly decreased AUC and TD in TF. However, increasing the KES amplitude beyond 1.0B *B*_TH_ resulted in no significant effect on the onset AUC and TD (figure 6(c)).

Conversely, increasing the KES amplitude leads to a significant increase in all three onset metrics measured in PF (*p* < 0.0001 for all metrics). In details, increasing the amplitude from 0.5*B*_TH_ to 1.0 *B*_TH_ significantly increases the onset peak-force, AUC, and TD in PF, but further increasing it within the tested range of amplitudes has no significant effect on the metrics measured in PF (figure 6(c)).

Increasing the KES frequency significantly reduced all three metrics observed in TF (figure 6(d)), Repeated measures ANOVA, *p* < 0.001 for all metrics). Only an increase in frequency from 15 kHz to 20 kHz showed no significant effect on TD. Increasing the KES frequency from 10 kHz to 20 kHz resulted in an average diminution of 56% (std = 62%) in AUC and 55% (std = 57%) in TD. The decrease in peak-force was less pronounced, with an average of 23% (std = 22%).

Increasing the KES frequency also resulted in significant reduction of the AUC and TD in PF, but was found to have no significant effect on the onset peak-force (figure 6(d)).

### 3.5. Muscle fatigue

**Figure 7.**
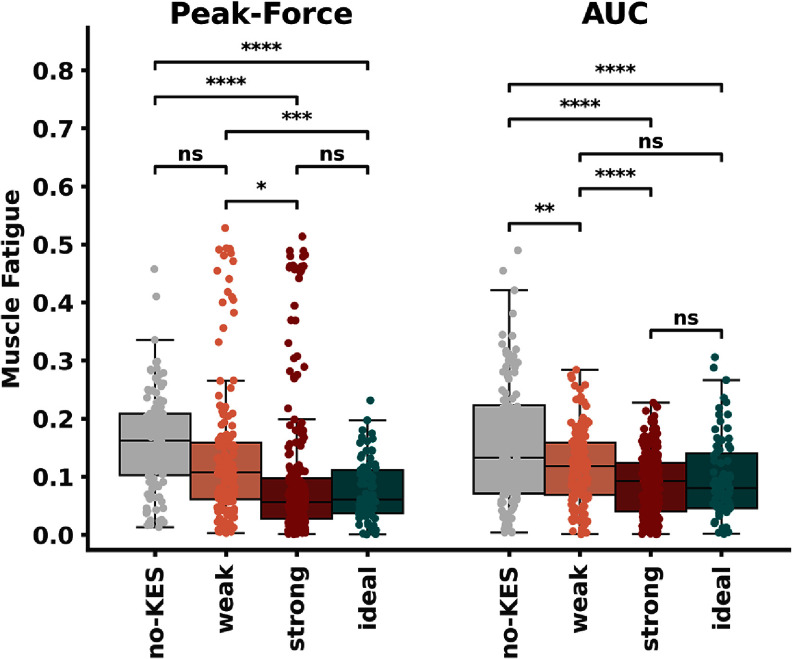
Muscle fatigue evaluation. Derived from peak-force and AUC during KES neural block ‘no-KES’, weak-block, strong-block and ideal-block scenarios are described in section 1.2. Significance is evaluated with a repeated measures ANOVA followed by a Bonferroni Post-hoc Test ns: non-significant * p < 0.05 ** p < 0.01 *** p < 0.001 **** p < 0.0001.

Muscle fatigue evaluated during *weak-* and *strong-block* conditions, as well as during the two control scenarios *no-KES* and *ideal-block* is shown in figure 7.

The muscle fatigue measurement scenario (*weak-block, strong-block, no-KES,* or *ideal-block*) significantly impacts muscle fatigue, as assessed by peak force and AUC measurements (Repeated Measures ANOVA, *p* < 0.0001 for both).

In the *no-KES* scenario, muscle fatigue resulted in a 17% decrease in peak force (std = 11%) and a 17% decrease in AUC (std = 13%). In the *weak-block* condition, muscle fatigue reduced peak force by 14% (std = 12%) and AUC by 12% (std = 8%). Fatigue was further reduced under the *strong-block* condition, with peak force decreasing by 10% (std = 12%) and AUC by 9% (std = 5%). The *ideal-block* scenario exhibited the least fatigue, with a decrease of only 9% (std = 10%) in peak force and 10% (std = 7%) in AUC.

Increasing the amount of KES neural conduction block leads to a significant reduction in observed muscle fatigue. Specifically, moving from the *no-KES* to the *weak-block* condition resulted in a 29% reduction in AUC (std = 15%), although the reduction in peak-force was not statistically significant. Muscle fatigue was further reduced by an additional 28% (std = 17%) in peak-force and 25% (std = 9%) in AUC when the KES block increased from *weak-block* to *strong-block*. Finally, the muscle fatigue measured by peak-force and AUC in the *ideal-block* scenario was not statistically different from that observed in the *strong-block* condition.

## 4. Discussion

This work demonstrates the feasibility of KES conduction block using LIFEs. We have also provided a comprehensive characterization of the block with LIFEs, based on parallels with activation (threshold, recruitment curves and selectivity indices) but also with specific aspects of the KES block (onset and muscle fatigue).

In particular, we demonstrated that it is possible to obtain a progressive and graded conduction block in a fascicle progressively, with a recruitment rate that can be modulated by adjusting the KES frequency. Moreover, we showed that interfascicular selectivity of neural conduction block can be achieved with LIFEs.

### 4.1. KES block thresholds

Blocking thresholds increase with increasing KES frequency. In the frequency range tested, this relationship is monotonic, which has been observed several times [29, 34, 49] and is also correctly predicted by simple computational models [35]. However, several studies reported a non-monotonic relationship between threshold and frequency, for slow fibers and for a KES frequency above 30 kHz [32, 50, 51]. Our experimental setup did not *a priori* allow us to observe this lack of monotonicity, since we focused on frequencies below 30 kHz and on the response of large, fast motor fibers [52]. In any case, non-monocity has not always been observed despite very similar experimental setups [31] and may be due to an experimental error (DC contamination) rather than a real physiological phenomenon [42, 53].

We also observed a quadratic relationship between blocking thresholds and KES frequency, while this relationship is generally considered to be linear [31, 54]. The resulting charge density, on the other hand, is nearly-constant in the tested range of frequency, but shows a local minimum and thus an optimum in terms of charge density and thus stimulation safety [55].

The study of normalized *B*_TH_ as a function of the KES half-period *T*_KES_ allows a direct comparison of block and activation thresholds. The comparison reveals a very high degree of similarity in the evolution of these two types of thresholds, despite profound differences in the underlying mechanisms. The effect on charge density is also comparable. It should be noted, however, that we define the activation threshold as the minimum current required to generate a muscle twitch, i.e., to recruit only a small pool of motor fibers, whereas the KES block threshold is defined as the minimum KES current required to completely block the muscle twitch, i.e., to block the entire pool of activated fibers.

As a starting hypothesis, we assumed that, as with activation, LIFEs and, more generally, intrafascicular electrodes allow for lower block thresholds than extrafascicular electrodes due to the closeness to the fibers. This hypothesis is supported by the fact that, as for activation, *in-silico* studies showed that *B*_TH_ increases with the electrode-axon distance [18, 35]. However, we measured block thresholds of the same order of magnitude or even higher than those observed with extrafascicular cuff-like electrodes [33, 37, 56].

At this stage, a first plausible explanation would be a radial misalignment of *aLIFE* and *bLIFE* during the implantation process. The pool of axons recruited by the *aLIFE* would therefore be geographically distant from the *bLIFE*, resulting in higher *B*_TH_. In the case of perfect alignment, axon tortuosity could also increase the distance between the *bLIFE* and the activated axons. However, the inter-LIFE distance is relatively small (<10 mm) and thus fibre tortuosity may be inconsequential at such short distances [57].

Also, the only other two studies demonstrating KES block with intrafascicular electrodes also showed high block thresholds of the same order [58, 59]. This suggests that this phenomenon is not specific to LIFEs, but rather to intrafascicular electrodes in general. We note that, overall, a limited amount of data is available on KES block with intrafascicular electrodes. A second hypothesis is discussed in section 3.5.

These high *B*_TH_ values raise concerns about charge density. In the case of LIFEs, the charge density limit for Pt–Ir electrode is about 100*μ*C cm^−2^ [22]. The observed maximum charge density varies between 74*μ*C cm^−2^ (at 10 kHz) and 70*μ*C cm^−2^ (at 30 kHz) and thus remains below the safety limit value. Only one measurement at a frequency of 5 kHz resulted in a threshold above the critical value (102 *μ*C cm^−2^). It is always possible to increase the size of the active electrode area to reduce the injected charge density and move away from the critical threshold of 100 *μ*C cm^−2^.

It is necessary to be aware that these safety limit values were historically established for conventional stimulation (e.g., monophasic and biphasic pulses) at frequencies much lower than KES, typically of 50 Hz [55, 60]. To date, it is unclear whether these values are relevant for KES stimulation, and it seems necessary to clarify it in view of the interest in this type of high-frequency stimulation [12].

The study on KES block using the Utah array by Dowden *et al* [58] reported a charge density of approximately 210 *μ*C cm^−2^, which is significantly higher than that what we reported with LIFES. However, while block thresholds are similar between extrafascicular electrodes and intrafascicular ones, the active surface area of extrafascicular electrodes is notably larger. Hence, charge density is typically roughly 1000 times lower with extrafascicular electrodes compared to intrafascicular ones [61, 62].

Previous studies have shown that with extrafascicular electrodes, the block threshold depends on their geometry and configuration [18, 63, 64]. Therefore, the KES block efficiency with LIFEs could also be enhanced by adjusting the geometry of the *bLIFE* (e.g., surface area) or its configuration (e.g., monopolar, bipolar, or tripolar).

### 4.2. Interfascicular selectivity and recruitment curves

In the mainstream approach to electrical stimulation for nerve fiber activation, intrafascicular electrodes differ from extrafascicular ones by having lower activation thresholds, while also providing enhanced selectivity for activating specific axon subpopulations. More specifically, electrodes such as LIFEs can push the interfascicular spillover far enough to recruit the population of an entire fascicle before recruiting fibers from a neighboring fascicle [45, 65].

In the context of using intrafascicular electrodes for neural conduction block with KES, we demonstrate here that, despite having block thresholds similar to those of extrafascicular electrodes, it is possible to selectively block one fascicle (TF) while minimizing spillover to an adjacent fascicle (PF). This limited interfascicular spillover is further improved by increasing the KES frequency, or in other words, increasing the KES frequency increases the interfascicular selectivity. For example, at a frequency of 20 kHz, the amount of block observed in the PF is less than 5%, despite a full block in the TF.

We also observed no blockade effect in the adjacent fascicle (PF) and for the 3 KES frequencies tested in 3 different data sets (2 rats). This may be due to the fact that, for these datasets, the *aLIFE-bLIFE* electrodes are implanted at opposite ends of the TF/PF border within their respective fascicles, i.e., they are placed far apart in a cross-sectional direction. Hence, it suggests that the interfascicular selectivity may be more pronounced when implanted in animal models with larger sciatic nerves, such as pigs.

The study by Dowden *et al* also demonstrated the possibility of selectively blocking a fascicle using Utah arrays [58], but was only achieved with 4 of the 55 electrodes. Conversely, once the experimental setup was stabilized with preliminary testing, we achieved a 100% success rate in eliciting conduction block with LIFEs. The study from Dowden *et al* suggested that implanting the KES block electrode as close as possible to the TF-PF branching zone could increase selectivity, as fascicle separation is more pronounced near this location. This could also further improve the KES interfascicular selectivity with LIFEs.

The KES block in TF versus amplitude consistently showed a gradual and progressive relationship. Similar results were also reported with extrafascicular cuff electrode [31]. The recruitment curves also showed that increasing KES frequency (or decreasing *T*_KES_) reduces the slope of recruitment. In other words, increasing KES frequency increases block controllability and improves interfascicular selectivity. Again, the parallel with activation holds, as decreasing pulse width also reduces the slope of the recruitment curves and improves controllability [66–68].

### 4.3. Onset response

The delivery of KES with LIFEs produces an onset response. This onset was short (<1 s) except when the KES frequency was set to 5 kHz, where we observed a prolonged onset (>5 s). We also observed an onset response longer than 1 s with higher KES frequency when the amplitude was below 0.5*B*_TH_.

We characterized the effect of KES amplitude (between 0.5 and 1.5*B*_TH_) and frequency (10, 15 and 20 kHz) on the onset response. We decoupled the onset produced by fibers activated in TF and in PF to estimate the onset response interfascicular spillover.

Increasing the KES amplitude has no effect on the TF onset peak force, but increasing the frequency does reduce it. To confirm that the effect was not solely due to fatigue, we put the frequency back to 10 kHz in several trials after testing at 20 kHz, and we confirmed that the onset peak force increases back again. Increasing the KES amplitude reduces the AUC and thus the onset response duration in TF. We observed a minimum of onset when the amplitude was close to *B*_TH_. The onset diminution is more pronounced with higher frequencies. Similar results were reported by Bhadra and Kilgore [34].

However, some studies show that increasing the KES amplitude beyond *B*_TH_ further reduces the onset [37, 39, 69]. We reasoned that the onset produced at *B*_TH_ in these studies is the result of the activation of the majority of nerve fibers, whereas in our study a smaller proportion of fibers are recruited at *B*_TH_, and increasing the KES amplitude beyond, recruits more fibers (un-recruited by the onset at *B*_TH_), thus resulting in an increased onset response.

Despite the delivery of KES via a LIFE implanted in the TF, an onset response was also observed in the PF. In other words, there is an onset response spillover in PF and which is more pronounced than that observed in conduction block. We observed that the maximum KES amplitude tested is well below the block threshold of most of the fibers in PF. The onset response tends to be stronger when the amplitude of the KES is below the *B*_TH_, which explains the presence of a strong onset in PF.

As in the TF, increasing KES frequency reduces onset duration in PF but has no significant effect on it is onset peak force. Increasing the KES amplitude increases the onset spillover, thus increasing the peak and onset duration. Further increasing the KES amplitude toward the *B*_TH_ in PF would eventually reduce the onset, but would increase the interfascicular conduction block spillover and thus be counterproductive.

Note that in the 3 data sets where we did not observe interfascicular conduction block spillover, we also did not observe onset spillover. It is therefore plausible that implantation in larger nerves, or in locations where the distance between fascicles is greater, will also eliminate onset spillover.

### 4.4. Fatigue and reversibility

The fatigue study demonstrates that, compared to fatigue induced solely by muscle activation, adding a period of KES block significantly reduces the observed fatigue. Furthermore, increasing the intensity of the block further mitigates muscle fatigue. This observation suggests that fewer muscle motor units are recruited during the block period, providing them with a time window for recovery. KES neural conduction block with LIFE therefore induces a true neural block at the fiber level, rather than causing a form of muscle tetany or co-contraction, which would not have reduced muscle fatigue.

We observed no significant difference between the muscular fatigue after a *strong-block* and an *ideal-block*, where neither muscular nor axonal fibers are activated at all. This suggests that the delivery of KES with LIFEs is not associated with additional neural fatigue, or only to a negligible extent. In overall terms, we observed almost instantaneous reversibility of KES, with a full force-recovery under a 1 s time window. We did not observe any *carry-over* phenomenon, which manifests itself in the maintenance of the conduction block several seconds or minutes after the cessation of KES delivery [12, 54].

However, it should be noted that this phenomenon is amplified by the duration of KES delivery [70], which in our case was very short, less than 4 s. Long-duration KES delivery with LIFEs should be performed to observe its effect on fatigue and reversibility. This *carry-over* phenomenon is also more pronounced in small fibers [30, 31], which this experimental setup does not allow us to observe. Further studies on KES delivery with LIFE will benefit from characterizing the *carry-over* phenomenon after extended KES delivery, with particular emphasis on the effect of KES frequency.

Lastly, this study on fatigue primarily focuses on the fatigue of large, fast-fatiguing motor units, as they are primarily recruited by electrical stimulation due to the reverse recruitment order mechanism [71], and they make the dominant contribution to the force profile, particularly in terms of peak force [72]. A study on the fatigue of smaller, fatigue-resistant fibers would benefit from an approach based on voluntary muscle contractions to bypass the reverse recruitment mechanism, combined with a more suitable recording strategy, such as EMG.

### 4.5. KES sub-threshold effect

In most trials, we observed a phenomenon of force amplification when delivering KES below the blocking threshold (<0.75*B*_TH_), i.e., the opposite of the effect of KES block typically reported (figure 11 of appendix 11, supplementary data). This force amplification effect vanishes with increasing KES amplitude, giving way to the conduction block phenomenon discussed in this study. It is important to emphasize that the force amplification manifested itself at the muscle twitch peak-force generated by stimulation on the *aLIFE* and not by the addition of an extra force, for example due to a long-lasting onset response. This amplification effect is visible on the recruitment curves as it results in a negative amount of conduction block at the lower end of the KES amplitude (see figures 4 and 5).

We hypothesize that there is a mutual coupling effect between the *aLIFE* and the *bLIFE*. This coupling results in facilitating fiber recruitment by the *aLIFE* when the *bLIFE* delivers a subthreshold KES stimulation. In other words, the KES delivery virtually lowers the activation threshold of some fibers during the block phase, resulting in more fibers being recruited by the *aLIFE* during this phase.

This phenomenon has not yet been reported by other studies, in which extrafascicular cuff-like electrodes were principaly used. Extracellular electrodes enable a greater distance between the activation and block electrode, and thus potentially limits this effect of coupling. Moreover, the activation electrode is typically used in a tripolar configuration, where the activation site of the electrode is surrounded by two grounds [61, 73, 74]. This configuration confines the electric field around the electrode [75, 76], which should further reduce the coupling effect. Finally, LIFEs and intrafascicular electrodes in general are implanted within the fascicle endoneurium, where the longitudinal conductivity is greater than that of the epineurium [77], which could also enhance this mutual electrode coupling phenomenon.

The coupling effect also adds a new hypothesis to explain the high *B*_TH_ of the intrafascicular electrode (see section 3.1). Indeed, delivering KES blocks some fibers recruited by *aLIFE* stimulation, but also facilitates the activation of another pool of motor fibers, counterbalancing the blocking effect. A full force block is therefore only possible when all motor fibers are blocked or when increasing the KES amplitude does not facilitate the activation of additional fibers.

This coupling effect remains an hypothesis for the time being, and will require proper *in silico* and *in vivo* studies to demonstrate it and analyze it.

### 4.6. Perspectives

The use of KES block with LIFEs is relevant for many therapeutical purposes as it brings benefits that are similar to activation: it allows the gradual, controlled block of a small group of fibers localized in a fascicle, without blocking fibers located in surrounding fascicles. The relevance of partial and/or selective KES block has been highlighted by Patel and Butera and classified as a requirement to facilitate the use of KES block clinically [12].

The use of intrafascicular electrodes leverages the somatotopic organization of the fascicles [78–81], which has been widely demonstrated in the context of electrical stimulation to activate fibers [65, 82–84]. This aspect can also be beneficially applied in the context of KES neural conduction block therapy with LIFEs. For example, the vagus nerve is divided into many fascicles and innervates many different organs, CNS structures, and other targets [9, 10]. Complete or indiscriminate conduction block of the vagus nerve can alter critical functions or cause significant side effects [11]. Similarly, a nonselective pudendal nerve block can cause various side effects such as incontinence or sexual dysfunction.

In this study we demonstrated that neural conduction block with LIFEs has a minimal interfascicular spillover an can result in a trully fascicle selective block. Future work will investigate the blocking of naturally generated neural activity, for example by generating tactile stimulation, to eliminate the need for an activating electrode and move one step closer to clinical application.

The KES onset-response remains a major limitation of the KES block. In particular, as with a non-selective block, uncontrolled onset-response can alter critical functions or cause significant side effects and may be prohibitive in certain applications. The use of LIFE reduces onset interfascicular spillover, thereby reducing the risk of onset activating unwanted pools of axons. Future work will also investigate KES modulation techniques and different electrode configurations to eliminate or reduce the onset response [39, 56, 69, 85, 86].

The issue of risks associated with prolonged KES neural conduction block remains an open question. Specifically, the relevance of charge density limit values must be evaluated with kilohertz-frequency continuous stimulation. The risk of long-term neural damage also requires assessment. However, it is important to highlight that chronic use of KES in humans has already been applied in the treatment of obesity and post-amputation pain [87, 88], which is promising in terms of safety and viability.

This work also demonstrated the feasability of combining the selective activation and block of fibers, opening a door to more complex stimulation strategies. Such a strategy could be used in people with upper or lower limb amputations to treat phantom pain with KES while providing somatosensory feedback using conventional stimulation, both via a single interface, such as a distributed intrafascicular multi-electrode lead system [89]. The similarities between fiber conduction block and fiber activation will also facilitate translation by using the same analysis tools.

This study investigates the potential for neural conduction block using LIFEs on motor fibers of the rat sciatic nerve. Such an approach could be applied to motor-related pathologies, including conditions like spasticity causing muscle overactivity [90], or the management of tremor [91]. We believe that such results can also be translated to different targets in the peripheral nervous system, and offer novel perspective to design future stimulation paradigms capable of alleviating specific symptoms while limiting side effects.

## Data Availability

The data that support the findings of this study are openly available at the following URL/DOI: https://zenodo.org/records/14755185.
